# Intradural disc herniation at L4/5 level causing Cauda equina syndrome: A case report

**DOI:** 10.1097/MD.0000000000019025

**Published:** 2020-02-14

**Authors:** Dawei Luo, Changbin Ji, Hui Xu, Hongyong Feng, Honglei Zhang, Kunpeng Li

**Affiliations:** Department of Orthopaedics, Liaocheng People's Hospital, China.

**Keywords:** cauda equina syndrome, diagnosis, intradural disc herniation, pathogenesis, surgical intervention

## Abstract

**Rationale:**

Intradural disc herniation has been documented rarely and the pathogenesis remains unclear. The region most frequently affected by intradural lumbar disc herniations is L4–5 level, and the average age of intradural disc herniations is between 50 and 60 years. Although magnetic resonance imaging is a valuable tool in the diagnosis of this disease, it is still difficult to make a definite diagnosis preoperatively.

**Patient concerns:**

In this report, we described a 58-year-old male patient who presented with intermittent pain of low back and radiating pain of the both lower extremities for 2 years as well as decreased muscle strength of the both legs and dysfunction of urinary and defecation for 1 month.

**Diagnosis:**

Lumbar disc herniation was diagnosed during the first clinical examination in the local hospital. Magnetic resonance imaging revealed a mass disc filling almost the entire spinal canal at the L4/5 level and a stalk connecting the mass to the intervertebral disc was detected in the sagittal T2-weighted image. The massive lesion caused cauda equina compression, resulting in dysfunction of urinary and defecation.

**Interventions:**

Considering the mass's volume, bilateral hemilaminectomy, and transforaminal lumbar interbody infusion were performed. During the surgery, we found a perforation in the ventral dura and major part of herniated disc was located in the intradural space through it. The disc was carefully dissected from the surrounding nerve roots and the ventral dura and then totally removed. The defect on the ventral dura was sutured to prevent cerebrospinal fluid leakage.

**Outcomes:**

The patient presented complete recovery of the radiculopathy and cauda equina syndrome and significant improvement of muscle strength of both legs at 12 months follow-up.

**Lessons:**

The diagnosis of intradural disc herniations is very difficult and mainly based on intraoperative and histopathological results. Surgical intervention is only effective method to manage this disease and to relieve symptoms and prevent severe neurological deficits.

## 1 Introduction

Intradural disc herniation (IDH) is defined as the extruded intervertebral disc through the dural matter and into the subarachnoid space, which is a very rare phenomenon in degenerative disc disease.^[1]^ Since the first case of posterior epidural migrated lumbar disc fragments was described by Dandy in 1942, there have been about 160 cases reported in the English-language literature until 2018. Currently, the pathogenesis of intradural disc herniation is unclear. Despite the advance of present neuroimaging techniques, such as computed tomography (CT) and magnetic resonance imaging (MRI), it is still difficult to make a definite diagnosis and confirm whether the disc herniation is located in the intradural space preoperatively.^[2]^ In most clinical cases, the accurate diagnosis is only confirmed during operation and according to the postoperative pathology. In the current report, we present a case of intradural migration of lumbar disc herniation at L4/5 level and discuss the clinical presentations, imaging features, treatments, and outcomes.

## 2 Case report

A 58-year-old male patient with a 2-year history of intermittent pain of low back and radiating pain of the both lower extremities was admitted to the hospital. He complained of increased and constant pain of the low back and both lower extremities that had started 1 month before. One month ago, he visited the local clinic and was diagnosed as lumbar disc herniation. He was given acupuncture and analgesic therapy, but the result was not satisfactory. Ten days ago, the patient complained of the aggravation of pain and decreased muscle strength of the both lower extremities, so he was admitted to the local hospital and defined as lumbar disc herniation with spinal canal stenosis by MRI. In these days, he also complained of dysfunction of urinary and defecation.

**Figure 1:**
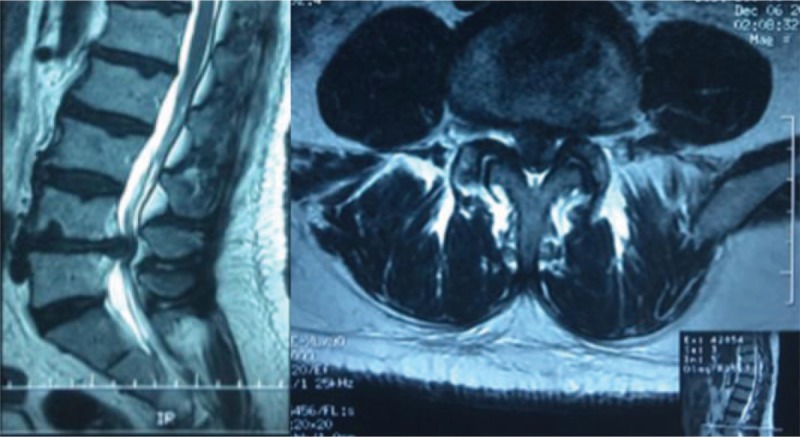
T2-weighted images in non-contrast sagittal(Left) and axial(Right) MRI demonstrating a huge isointense lesion, located at L4/5 level, which nearly compresses the entire spinal canal in the T2-weighted images.

**Figure 2:**
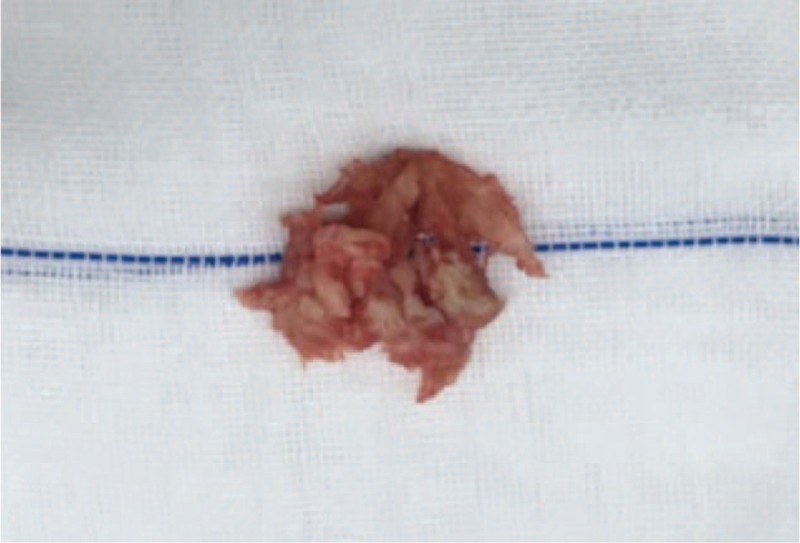
Intraoperative image demonstrating the lesion being similar to the degeneative interverbral disc.

Physical examination showed that hypesthesia of the posterolateral aspect of both lower extremities, weakness of both extensor hallucis longus and musculi hippicus (grade 3), negative Lasegue sign on the double limbs, decreased knee tendon and ankle reflex in both lower extremities, and dysfunction of bladder and bowel. Anterior–posterior radiographs demonstrated degenerative changes without scoliosis. CT scans showed herniated nucleus pulposus at the L4/5 level. MRI revealed a mass disc filling almost the entire spinal canal at the L4/5 level, causing cauda equina compression. The lesion was hyperdense on T2-weighted and hypointense T1-weighted images (Fig. 1). A stalk connecting the mass to the intervertebral disc was detected in the sagittal T2-weighted image. A herniated lumbar disc could be confirmed, but whether the disc herniation was located in the normally extradural space was suspected. Bilateral hemilaminectomy was performed. The adhesions between posterior longitudinal ligament (PLL) and the ventral dura was extremely resistant and it was very difficult to be separated through sharpless dissection. There was a perforation in the ventral dura and most part of herniated disc was located in the intradural space through the perforation. The disc was carefully dissected from the surrounding nerve roots and the ventral dura and then totally removed (Fig. 2). The defect on the ventral dura was sutured to prevent cerebrospinal fluid leakage after surgery. Pathologic examination confirmed that the mass was degenerated intervertebral disc. The patient presented complete recovery of the radiculopathy and cauda equina syndrome and significant improvement of muscle strength of both legs at 12 months follow-up. This patient has provided written informed consent for publication of the case.

## 3 Discussion

IDH is a rare condition, only accounting for 0.26% to 0.33% of all disc herniations and it occurs more often in the lumbar region.^[3]^ Oztürk^[4]^ reported that 92% of all IDH cases occurred in the lumbar level, with only 5% occurring in the thoracic level and 3% in the cervical level. Liu^[5]^ found that the level most frequently affected by IDH is L4–L5. The average age of onset of intradural disc herniations is between 50 and 60 years and males are 4 times more likely to be affected than females. Usually, IDH is frequently associated with symptoms worse than the regular disc herniation. Previous study reported that clinical manifestation of IDH in the cervical region shows a severe neurologic deficit such as Brown Sequard syndrome, incomplete or transient quadriparesis^[6,7]^ and cauda equina syndrome found in two-thirds of the patients in the lumbar region.^[5]^ This case in our report revealed intermittent pain of low back, radiating pain and decreased muscle strength of the both lower extremities, and dysfunction of urinary and defecation, combining with radiculopathy and cauda equina syndrome. Both the radiculopathy and cauda equina syndrome recovered to some extent postoperatively, but there was only slight improvement of muscle strength of the both lower extremities.

The pathogenesis of IDH still remains unclear. Migration of the disc fragment into the intradural space requires perforation of the annulus fibrosus, the PLL and the dura mater.^[8]^ Several reports presented that some factors were responsible for IDH, such as less epidural space caused by congenital or iatrogenic narrowing of the spinal canal, adhesions between the annulus fibrosus, PLL and dura mater, and vulnerability of the dura mater.^[8–10]^ Among these, the adhesions between the ventral dura and the PLL were thought as important predisposing factors. Tateiwa^[2]^ reported that these adhesions would be caused by some iatrogenic or congenital factors such as dural thickness, previous spine surgery and local trauma. Floeth and Herdmann^[11]^ believed that chronic inflammation, resulting from the degenerative disc pathologies, would favor the occurrence of these adhesions and lead to erosion process with thinning of the dura. Ducati^[12]^ demonstrated that the dura mater and PLL were anatomically closest at the L4/5 level, and such adhesions occurred more often at this level, which may explain the higher incidence of IDH in this region. However, Pan^[13]^ reported another possible mechanism that the herniated disc, as a “foreign body” to the spinal canal, would activate autoimmunoreactivity, and he presumed that biochemical factors may play an important role in the pathogenesis of IDH. In our case, the adhesion between PLL and ventral dural was very severe and the dura mater was very fragile in this region. We speculated that many factors including congenital canal stenosis and dura fragility, history of spine surgery, trauma, and disc degeneration lead to the chronic process of inflammation and immunoreactivity, and during this process long-term mild irritation or a sudden force caused the prolapsed disc into the intradural space through the erosions on the dura.

Though MRI, a gold standard in neuroimage, has been widely used, it remains difficult to definitely identify IDH preoperatively, because it is a rare pathology and easily confused with other spinal lesions, such as intradural tumors, arachnoid cyst, metastasis, and subdural abscess. Generally, a disc fragment demonstrated hypointense on T1-weighted images, and most hyperintense on T2-weighted images.^[14]^ There were some indirect indicative signs to identify perforation of the dural sac on MRI imaging. Choi^[15]^ thought that useful MRI findings such as abrupt discontinuity of PLL and “hawk-beak sign”, which was a sharp compressing lesion with a beak-like appearance to the dural sac on T2-weighted axial images, indicated the potential presence of IDH in the lumbar spine. Sasaji^[16]^ demonstrated that the “Y-sign” on T2-weighted sagittal images of MRI was a characteristic finding of extra-arachnoid lumbar IDH and useful to make accurate diagnosis. Mailleux^[17]^ reported a specific MRI sign, “crumble disc sign”, contribute to make definite diagnosis for the intradural localization of the disc herniation. The most common radiological signs seen in IDH is the rim enhancement on the gadolinium-enhanced MRI, which was thought to be the result of the infiltration of the vascular granulation tissue into the lesion.^[18]^ However, this sign was not specific for IDH. In the case we presented, the “hawk beak” sign and “Y” sign were not shown as mentioned above, so the definite diagnosis was not obtained preoperatively. Recently, Crivelli^[19]^ proposed that three-dimensional high-resolution constructive interference in steady state sequence should be considered when IDH was suspected on conventional MRI, which may accurately depict IDH and optimally guide the surgical approach.

Surgical intervention is the only effective method to treat the disease. Posterior decompressive procedure is a popular surgical approach in removal of the intradural disc sequestrum. Kobayashi^[20]^ reported that discectomy with interbody fusion was performed to completely remove the disc and prevent recurrence. We adopted bilateral hemilaminectomy and transforaminal lumbar interbody infusion considering large size of lesion, and removed the disc fragments completely. During the surgery, we found the major part of herniated disc was located in the subarachnoid space and the rest in the epidural area, which may be an intermediate process of herniated disc moving toward the intradural region. This could confirm the presumed theory that the adhesions between the PLL and the ventral wall of dura mater due to congenital union, chronic mechanic irritation or inflammation caused the fragility in the dura mater and PLL, and subsequently, the herniated disc perforated dura mater by a sudden force.^[10,21]^ Kim^[22]^ performed transforaminal endoscopic technique to manage intradural disk herniation at an L2-L3 level and obtained good clinical outcomes. Anterior lumbar interbody fusion was another alternative in the treatment of IDH that can avoid unnecessary retraction of the nerve root and dura. However, it is limited into patients who was made definite diagnosis preoperatively. Epstein^[23]^ reported in a review that unmanaged durotomies were able to result in cerebrospinal fluid fistula, pseudomeningocoele, meningitis, and postural headache. Several methods were used to manage the dura defects in case reports, such as closure with artificial membrane and an autogenous graft, or direct suture.^[24]^ To minimize injuries to nerve roots, surgical microscope was recommended to provide a very clear visual field and allow a careful dissection of disc fragments when performing the removal of disc material and closure of the dura rupture.^[25]^ In this case, the dissection of disc fragments and direct suture of the dura defect were carefully performed with the aid of microscope. A posterior transpedicular fixation system was implanted to maintain the stability of spine in addition to lumbar interbody fusion. The patient presented complete recovery of the radiculopathy and cauda equina syndrome and significant improvement of muscle strength of both legs at 12 months follow-up.

## Author contributions

**Conceptualization:** Kunpeng Li.

**Data curation:** Dawei Luo, Hui Xu, Hongyong Feng.

**Investigation:** Changbin Ji, Hongyong Feng, Kunpeng Li.

**Project administration:** Changbin Ji, Honglei Zhang.

**Resources:** Honglei Zhang.

**Writing – original draft:** Dawei Luo.

**Writing – review & editing:** Hui Xu, Kunpeng Li.
